# Deconstructing mastery in colorectal fluorescence angiography interpretation

**DOI:** 10.1007/s00464-022-09299-3

**Published:** 2022-05-11

**Authors:** Jeffrey Dalli, Sarah Shanahan, Niall P. Hardy, Manish Chand, Roel Hompes, David Jayne, Frederic Ris, Antonino Spinelli, Steven Wexner, Ronan A. Cahill

**Affiliations:** 1 UCD Centre for Precision Surgery, School of Medicine, Catherine McAuley Centre, University College Dublin, 21 Nelson St, Phibsborough, Dublin 7, D07 KX5K Ireland; 2 UCL Division of Surgery and Interventional Sciences, WEISS Centre, University College London, London, UK; 3 Department of Surgery, Amsterdam UMC (AMC), University of Amsterdam, Amsterdam, The Netherlands; 4 Leeds Institute for Medical Research, University of Leeds, Leeds, UK; 5 Service of Visceral Surgery, Geneva University Hospitals and Medical School, Geneva, Switzerland; 6 Department of Biomedical Sciences, Humanitas University, Via Rita Levi Montalcini 4, 20090 Pieve Emanuele, MI Italy; 7 IRCCS Humanitas Research Hospital, via Manzoni 56, 20089 Rozzano, MI Italy; 8 Department of Colorectal Surgery, Cleveland Clinic Florida, Weston, FL 33331 USA; 9 Department of Surgery, Mater Misericordiae University Hospital, Dublin, Ireland

**Keywords:** Indocyanine green, Fluorescence, Angiography, Digital, Quantitative

## Abstract

**Introduction:**

Indocyanine green fluorescence angiography (ICGFA) is commonly used in colorectal anastomotic practice with limited pre-training. Recent work has shown that there is considerable inconsistency in signal interpretation between surgeons with minimal or no experience versus those consciously invested in mastery of the technique. Here, we deconstruct the fluorescence signal patterns of expert-annotated surgical ICGFA videos to understand better their correlation and combine this with structured interviews to ascertain whether such interpretative capability is conscious or unconscious.

**Methods:**

For fluorescence signal analysis, expert-annotated ICGFA videos (*n* = 24) were quantitatively interrogated using a boutique intensity tracker (IBM Research) to generate signal time plots. Such fluorescence intensity data were examined for inter-observer correlation (Intraclass Correlation Coefficients, ICC) at specific curve milestones: the maximum fluorescence signal (F_max_), the times to both achieve this maximum (T_max_), as well as half this maximum (T_1/2max_) and the ratio between these (T_1/2_/T_max_). Formal tele-interview with contributing experts (*n* = 6) was conducted with the narrative transcripts being thematically mapped, plotted, and qualitatively analyzed.

**Results:**

Correlation by mathematical measures was *excellent* (ICC0.9–1.0) for F_max_, T_max_, and T_1/2max_ (0.95, 0.938, and 0.925, respectively) and moderate (0.5–0.75) for T_1/2_/T_max_ (0.729). While all experts narrated a deliberate viewing strategy, their specific dynamic signal appreciation differed in the manner of description.

**Conclusion:**

Expert ICGFA users demonstrate high correlation in mathematical measures of their signal interpretation although do so tacitly. Computational quantification of expert behavior can help develop the necessary lexicon and training sets as well as computer vision methodology to better exploit ICGFA technology.

**Supplementary Information:**

The online version contains supplementary material available at 10.1007/s00464-022-09299-3.

Indocyanine green fluorescence angiography (ICGFA) has been rapidly adopted across many surgical disciplines [1]. It has become especially established in colorectal anastomosis assessment protocols during laparoscopic- and robotic-assisted surgeries due to its potential benefits in diminishing anastomotic leakage and its clinical [2], oncologic [3], and economic [4] impacts. Despite such momentum, including ongoing multinational randomized trials [5], variability in its clinical deployment and study outcomes exists [6, 7]. Previous work by this research collaborative demonstrated considerable inter-user variability in ICGFA interpretation, especially among inexperienced users [8]. In contrast, experienced users displayed better correlation in their decisions regarding geographical determination of colonic proximal transection levels. Better understanding of these observations could facilitate improved utilization of this technology allowing its true value to be extracted by all users.

Therefore, here we follow on the initial observer variability discovery work with a focused investigation of the interpretation and cognitive processes occurring at expert user level in that study via semi-structured interviews as well as detailed quantitative analysis of observed video signal interpretation. The purpose of our study was to evaluate factors underlying the better correlation seen between experts and to establish whether this occurs due to conscious act in order to inform how best to advance ICGFA interpretation among others.

## Methodology

### Prior study

This research [8] (performed with institutional ethics approval Mater Misericordiae University Hospital, Dublin, Ireland—1/378/2092) involved the presentation of an archived set of edited ICGFA video sequences (*n* = 14, 9 showing perfusion signals at the time of proximal colonic transection, five showing anastomotic/small bowel perfusion) obtained during routine laparoscopic colorectal surgery to both experienced (i.e., those who are highly active clinically and academically in the field of ICGFA, *n* = 6) and inexperienced (i.e., those with minimal or no ICG exposure whether clinically experienced or inexperienced, *n* = 34) observers via an innovative video display and annotation platform (Mindstamp: The Interactive Video Platform www.mindstamp.io). All ICGFA transection perfusion videos demonstrated the white-light tissue appearances as well as the near-infrared (NIR) appearances and an overlay, false-colored view of the NIR signal on the white-light image simultaneously using the Pinpoint Endolaparoscopic Near-infrared system (Stryker^©^). Using Mindstamp, observers were able to record their interpretations of the fluorescence signal of the proximal colonic segment directly onto the video segment, indicating their choice of stapler positioning across the colon based on their interpretation of the fluorescence signal. This allowed comparison of interpretations between experienced and inexperienced users and showed significant differences in interpretation between the two groups with experienced users showing higher levels of agreement in signal interpretation.

For this study, the specific sites of transection level selection in four expert-annotated videos from the first study were analyzed to both generate and correlate fluorescence dynamic signal patterns as a time series of intensity to understand better the patterns of recognition of the experienced observers. The four videos chosen were those selected as most optimal for computational assessment regarding minimal camera movement and instrument intrusion. In addition, the same expert participants were interviewed to understand their conscious thought processes in interpreting dynamic fluorescence signals.

**Fig. 1:**
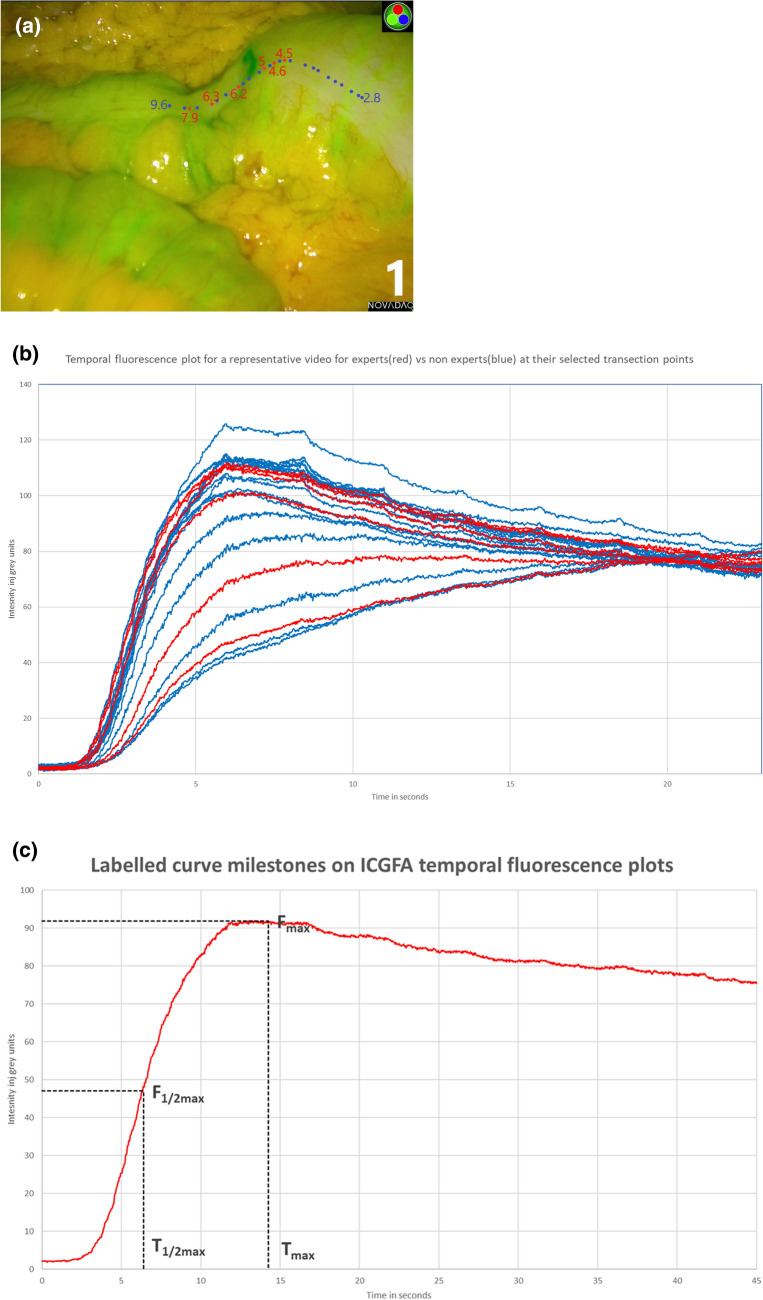
**a** Transection annotation points on the ascending colon (made on a video recording of anastomotic preparation site during right hemicolectomy) showing experts (red) and inexperts (blue) on a fused NIR and White-light ICGFA still image from the ICGFA video (from [7]) and ascending numbers represent increasing distance (proximal to distal) in mtu (“measuring tool units”) generated using Snip and Sketch, Microsoft, (NM, US) [8]. **b** Quantitative intensity fluorescence time plots charted from these sites, including here, for illustrative purposes, those of inexpert users. **c** Specific curve milestones from an idealized plot selected for the purposes of comparative analysis (Color figure online)

For fluorescence signal analysis, videos (30 frames per second) of four intraoperative ICG angiograms used in the prior study were analyzed using a boutique intensity tracker (IBM Research Ireland [9]) to generate quantitative plots over time of the fluorescence signal at the transection points selected by expert users (*n* = 6) (see Fig. 1) (camera movement and instrument intrusion compromised continuous transection point machine-based tracking in the remaining five videos from the prior study and so these were not useable in this work). User-selected regions of interest (ROI) in the white-light view were digitally tracked via surface features and concurrent fluorescence intensity was simultaneously quantified in the corresponding regions of the raw near-infrared feed.

**Fig. 2:**
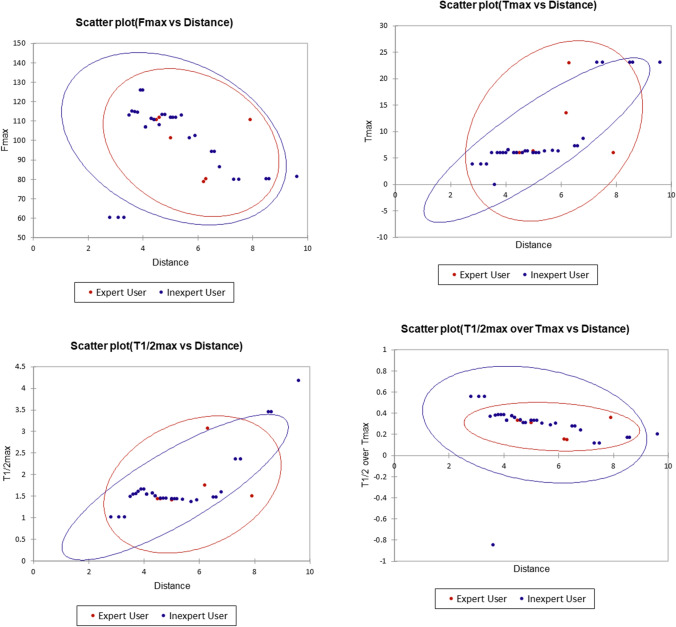
Quantitative intensity fluorescence plots from expert-annotated video related to transection level (same video as Figure One) are shown here for data illustrative purposes. Plots are tagged for milestones (F_max_, T_max_, T_1/2max_ and T_1/2max_/T_max_) in comparison against those plots associated with inexperts (blue) on the same video. Scatter temporal fluorescence plots with Chi-squared confidence ellipses (XLSTAT v2021, Addinsoft for Microsoft Excel) for both experts and inexperts are shown with respect to geographical transection point for each predefined curve milestone (Color figure online)

The generated fluorescence intensity data were recorded on Microsoft Excel v2012 (NM, USA). Specific curve milestones identified previously as those most clinically meaningful indicators of appropriate ICGFA perfusion signal [10, 11] were identified and tabulated as metadata. These milestones were the maximum fluorescence signal (F_max_), the time required for it to rise to fifty percent of the maximum fluorescence intensity (T_1/2max_), and the time to achieve the full peak (T_max_), as well as the ratio of the two (T_1/2_/T_max_) (see Fig. 2). Correlation of these parameters among experts was determined by Intraclass Correlation Coefficients (ICC: two-way mixed model, absolute agreement, single measures) [12] calculated using SPSS v26 (IBM, NY, US). To illustrate expert correlation in comparison to inexperienced user data dispersion, this exercise was also carried out for inexperienced users (*n* = 34) for one video and charted as scatter plots, with Chi-squared confidence ellipses charted to visually demonstrate expert/inexpert clustering of curve features.

### Experienced user interviews

To understand any conscious viewing strategy applied by each experienced user in the study, each underwent formal interview conducted by teleconference using a template of open and closed questions relating to specific and general uses of ICG fluorescence with particular reference to signal interpretation (see supplementary information). Responses were recorded, transcribed, and qualitatively assessed via thematic analysis [13] (in short, ideas, concepts, opinions, statements, and descriptions were identified, coded, and grouped by emergent themes and mapped graphically with overarching categories as the stem and individual terms as the roots). In addition, a word cloud was plotted to give a visual summary of the main terminology used following the removal of definite and indefinite articles, determiners, pronouns, names, conjunctions, interjections, adjective pre-modifiers, collective nouns, and pre-prepositions, and prepositions using dedicated software www.wordclouds.com (Zygmomatic, Vianen, The Netherlands). Although not academically quantitative, this method of graphical representation groups commonest words used by size with increasing font size indicating more frequently used terms as a simple visual means of summarizing common words and terms.

## Results

Six thousand forty data points per expert (*n* = 6) were synthesized from the ICGFA videos (*n* = 4).

### Fluorescence signal analysis

Once plotted, the quantitative fluorescence curves were analyzed regarding F_max_, T_max_, T_1/2max_, and T_1/2max_/T_max_ (see Table 1 and Fig. 1). Application of inter-observer comparative statistics revealed *excellent* (0.9–1.0) levels of correlation among these experienced users regarding peak intensity (F_max_ 0.925) and chronological flags (T_max_ 0.938 and T_1/2max_ 0.925) and *moderate* (0.5–0.75) correlation for the T_1/2max_/T_max_ ratio (0.729).

**Table 1:** Compound table showing comparative data related to temporal fluorescence milestones (generated as metadata from quantitative ICGFA fluorescence time plots at the, respectively, selected expert colonic geographical transection points across four videos [8]) reported per video and per expert (mean ± standard deviation)

Temporal fluorescence milestones	Comparison between videos		Comparison between experts			
	Video: Mean ± s.d		Expert: Mean ± s.d		Intraclass correlation coefficient	
					Intraclass correlation	95% Confidence interval
F_max_			1	98.10 ± 35.28	0.925 (excellent)	0.740–0.994
	1	99.14 ± 16.74	2	92.78 ± 36.22		
	2	147.83 ± 4.68	3	97.40 ± 34.68		
	3	68.39 ± 3.20	4	103.22 ± 36.43		
	4	80.51 ± 7.05	5	104.14 ± 38.91		
			6	98.18 ± 34.84		
T_max_			1	25.65 ± 13.11	0.938 (excellent)	0.777–0.995
	1	9.69 ± 6.78	2	27.01 ± 24.07		
	2	19.70 ± 0.96	3	26.05 ± 25.03		
	3	15.37 ± 1.57	4	26.37 ± 24.78		
	4	59.53 ± 7.34	5	26.19 ± 24.82		
			6	25.16 ± 25.46		
T_1/2max_			1	3.83 ± 2.61	0.925 (excellent)	0.741–0.994
	1	1.76 ± 0.57	2	4.09 ± 3.91		
	2	3.10 ± 0.16	3	3.98 ± 3.88		
	3	1.69 ± 0.12	4	4.85 ± 5.51		
	4	11.00 ± 2.30	5	4.82 ± 5.42		
			6	4.76 ± 5.49		
T_1/2max_/T_max_			1	0.32 ± 0.15	0.729 (moderate)	0.343–0.976
	1	0.28 ± 0.08	2	0.30 ± 0.09		
	2	0.47 ± 0.05	3	0.32 ± 0.06		
	3	0.26 ± 0.05	4	0.35 ± 0.12		
	4	0.37 ± 0.06	5	0.36 ± 0.12		
			6	0.40 ± 0.09		

Intraclass correlation coefficients between experts (with poor agreement set at < 0.5, moderate at 0.5–0.75, good at 0.75–0.9, and excellent at 0.9–1.0) are shown [12]. All the ICC correlation coefficients reported above (F_max_, T_max_, T_1/2max_, and T_1/2max_/T_max_) have a significance of *p* < 0.001

### Expert interview data

**Fig. 3:**
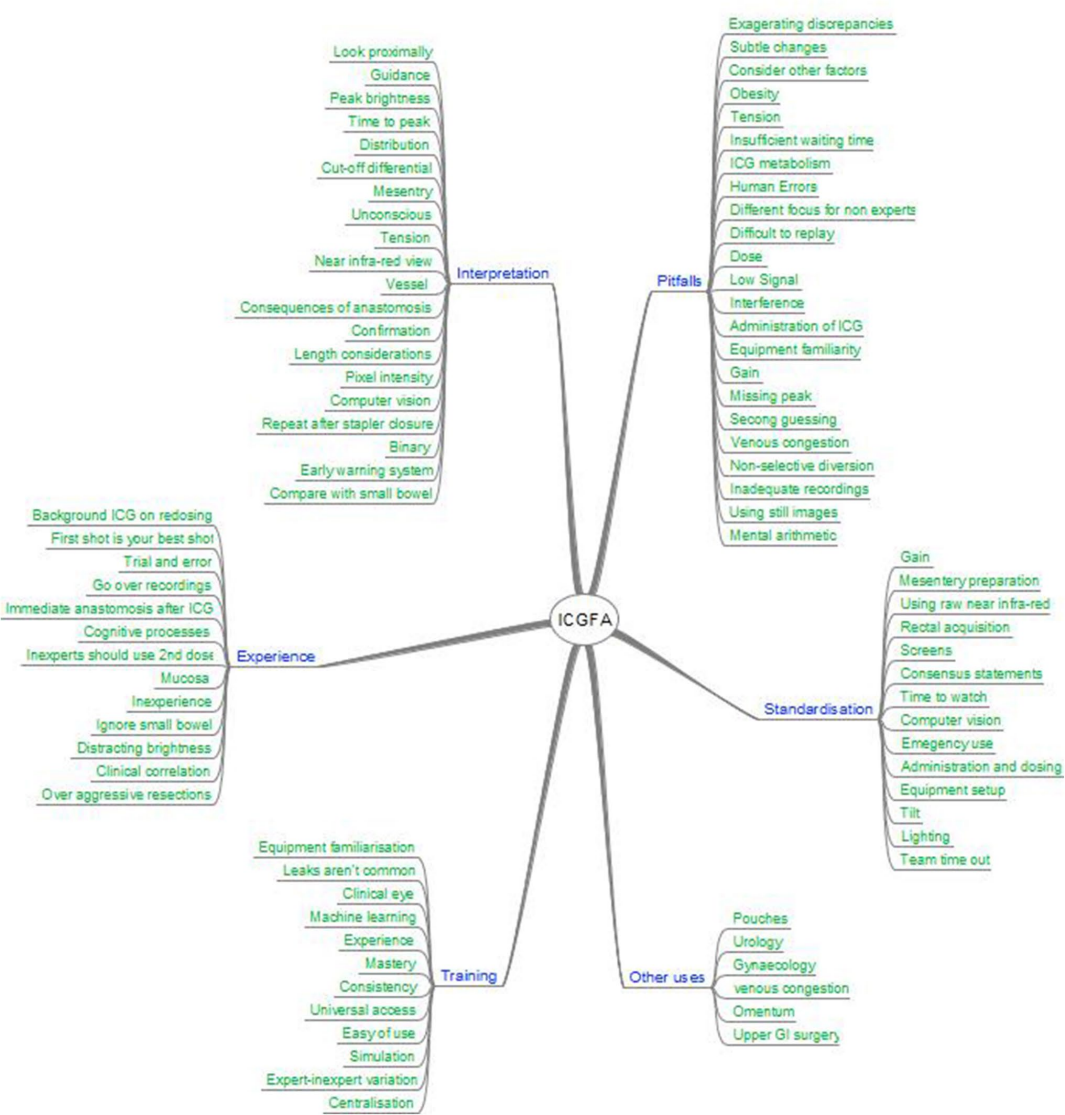
Thematic map accrued via qualitative analysis [13] of expert interview transcripts (*n* = 6). Here expert interview responses were transcribed, and ideas, concepts, opinions, statements, and descriptions were identified, coded, and grouped by emergent themes (the graphical map shows overarching categories as the stem and individual terms as the roots)

**Fig. 4:**
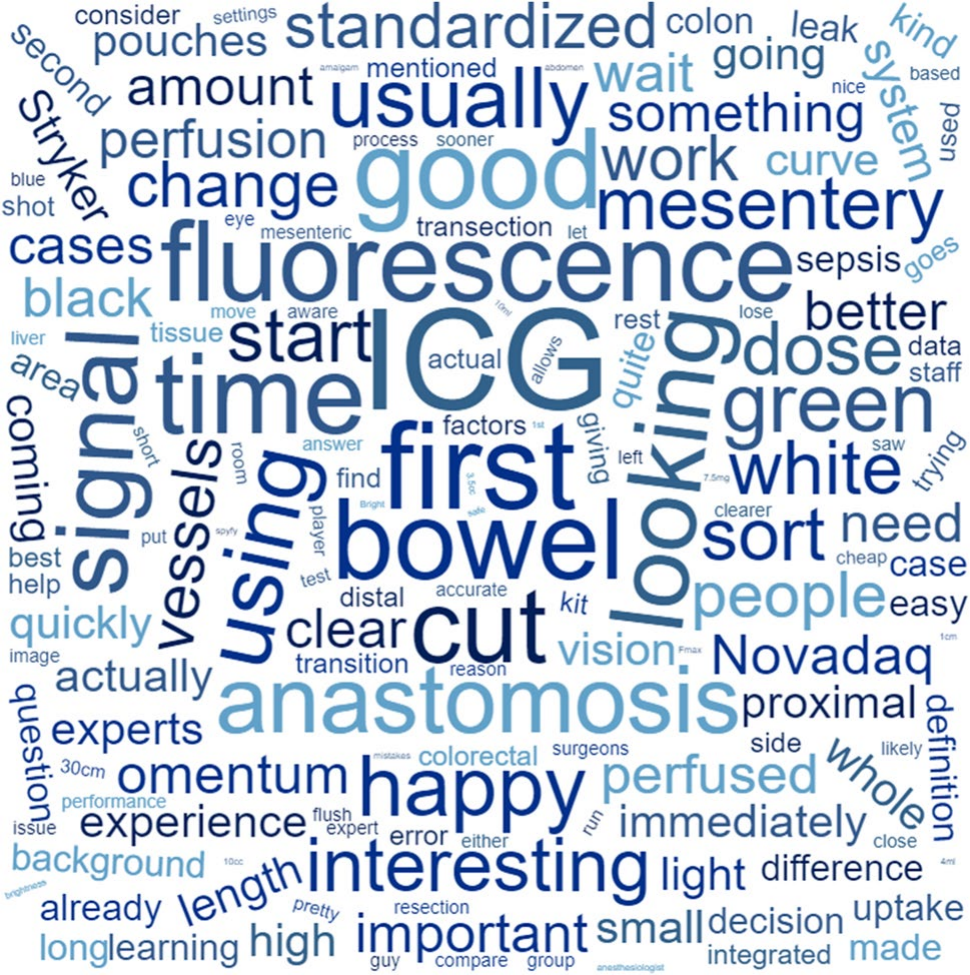
Word plot (www.wordclouds.com, Zygmomatic, Vianen, the Netherlands) of expert responses to survey questions (see supplementary material). Definite and indefinite articles, determiners, pronouns, names, conjunctions, interjections, adjective pre-modifiers, collective nouns, and pre-prepositions and prepositions have been removed. Font size reflects how commonly the term was used provided a quantitative display of most used words, while color and direction are purely for ease of artistic representation

Structured discussion with each experienced observer demonstrated that each use ICGFA as a confirmatory rather than directive measure with respect to sufficiency of bowel perfusion (i.e., a specific area is being visually evaluated for a reasonable perfusion signal) with a focus on inflow alone and each employs a deliberate interrogative viewing strategy to do so. Furthermore, all advocated a conscious systematic approach to learning including preclinical simulation as opposed to operative experiential learning although none had done so themselves or has such a facility in their departments. The experts however described their exact methodology in a variety of ways without common precise terminology (see Figs. 3, 4). Furthermore, more frequent comments were made on methods of ICG set-up rather than its interpretation. While intraoperative redosing and replaying the video were not recommended, a common theme of an ICG ‘time out’ (where the whole theater staff pause to appreciate the ICG signal) was prevalent. There were mixed opinions on the use of composite screen viewing (four utilizing this view) versus viewing the near-infrared signal appearances alone (*n* = 2) with the former espousing the benefit of simultaneous interpretation of white-light image appearances. Minimalist systems with fewer options for signal output manipulation were favored. Among considerations other than on-screen fluorescence signal, advocacy for standardization to fixed dosing and administrative method protocols was common with less regard for exact dosing, titration to weight, or ideal signal performance. With respect to inflow signal interpretation, homogeneity of distribution and maximal brightness were prioritized criteria although there was otherwise divergence in opinions re the other proposed criteria. A single expert admitted to using the chronology of the fluorescence as a discrete transection modifying criteria, while two others actively expressed that they did not feel this to be important.

## Discussion

Intraoperative decision-making is an empirically learned skill which involves interpretation of visual appearances through judgment based on knowledge and experience. As a dynamic signal, interpretation of ICGFA also requires qualitative interpretation which appears to improve with experience although at present this lacks overt standardization [14] with consensus groups yet to define ideal parameters [15]. This heterogeneity in practice and techniques is also reflected in research studies with some either disregarding [16] or variably appreciating [6, 7] the chronology of the fluorescence intensity. Technical considerations such as ICG-tissue interaction and NIR system performance (including brand specific on-screen display) [17–20] may further impair correct ICGFA perfusion signal interpretation. All of this means that correct interpretation of ICGFA signals may require some experience. Furthermore, the limitation of purely visual analysis drives confirmatory-focused signal appreciation as opposed to full-field exploratory interrogation (i.e., people can only easily look at one region at a time). This work follows on from an initial study [8] that examined if there are differences between experts and inexperienced users regarding ICGFA interpretation using a curated archive of actual surgical cases [8] and uses the same dataset. This prior work showed better levels of ICC among experts (0.753 good) vs inexperts even if clinically experienced (0.613 moderate) and indeed further additional work has shown similar discrepancy exists with regard to ICGFA signal interpretation in upper gastrointestinal surgery [21]. This present work focused on examining how consistent same experts were with each other in reaching their interpretations and whether any such consistency was more explainable either their verbalized descriptions or via mathematical deconstruction of the dynamic fluorescence signal being viewed.

### Main findings

Our experienced users showed excellent tacit agreement in their selections of bowel locations for proximal transections and their selection points were found to have highly similar curve profiles suggesting a common acquired ability to similarly dissect the dynamic signal being observed and flag the most relevant clinical location in a highly concordant fashion. Specifically, the peak intensity (F_max_), the time to achieve this zenith (T_max_), and the threshold period at which half this incline has been achieved (T_1/2max_) demonstrated excellent correlation. Interestingly, compound metrics identified experimentally as prognostically sensitive for anastomotic leakage (T_1/2max_/T_max_) [10] resulted in only moderate correlation. However, despite expert signal interpretation showing great consistency, this was neither consciously appreciated nor reflected in agreement in the recorded interviews. This perhaps should not be too surprising as mastery can often struggle to explain itself in the absence of objective deconstruction and standardized terminology.

### Implications for practice

This study’s findings do suggest a method for explaining to others how best to extract the most useful information from ICGFA through its observation, perhaps in training libraries that users can study as they accrue their own clinical experience showing patterns of ICGFA signaling at different areas across a screen with indication of where an expert surgeon would choose to affect a clinical decision. Furthermore, the digital deconstruction of ICGFA methodology it uses could also be used for post hoc reflection on trials and experiences most especially those with equivocal or negative results [16]. This study’s observations also of course suggest a role for real-time quantitative data synthesis, analysis (including statistical correlation), and display to supplement an observer’s own interpretation especially early in their learning curves. While mathematical flagging of the flow patterns post hoc on operative video recordings could be useful for training curriculums, “on-the-fly” compound computation of fluorescent signals is needed for intraoperative decision support [22]. However the accuracy of such signal plot may be confounded by interpatient [23], variations in ICG pharmacokinetics and pharmacodynamics, as well as subject to influence by current NIR system inconsistencies across the field of interest (such systems have been primarily designed for image display for surgeon interpretation rather than being precision quantification tools). Relative, signal representation e.g., identifying the most distal bowel region displaying at least 80% of the maximum fluorescence intensity observed on-screen could be a clinically valuable near-term achievement. However, progressing onto computational assessment requires careful videography, avoiding instrument intrusion and excessive movement which hampered analysis of certain videos from our previous series. Digitalizing surgical decision-making processes is interlinked too with ethical, medico-legal, moral, and privacy issues that must also be taken into consideration [24].

### Implications for research

This work so prompts further study and informs any future investigators regarding potentially useful parameters to investigate in future collaborative, multicentered work to cultivate greater datasets and a body of evidence to guide best practice and aid in surgical simulation, training, and research.

### Other considerations

This work is of course limited by the relatively small numbers involved but it does enable larger studies now to be performed to test and tease out these findings more robustly and indeed determine the usefulness of interpretation training for surgeons beginning their ICGFA use. It is possible too that actual intraoperative interpretations (which include many other experiential prompts to decision-making and likely greater focus in concentration) differ from those made when watching isolated video alone and this also needs greater understanding (likely best through studies of inter- and intra-observer interpretations in theater versus out of theater).

In conclusion, ICGFA interpretation appears to standardize with experience although at present such expertise is tacitly acquired. Computational charting of the fluorescence signal into quantitative ICGFA plots over time seems able to provide a mathematical way to understand clinical mastery in interpretation potentially allowing introduction of a standard lexicon to verbally articulate the peaks and troughs of these visible intensity fluctuations. More complete understanding of expert interpretation may also inform machine-based analysis methods to automatically provide ICGFA interpretation in the future although further work is necessary to achieve this goal.

## Supplementary Information

Below is the link to the electronic supplementary material.

Supplementary file1 (DOCX 13 kb)
