# Supplementary material for: Deconstructing mastery in colorectal fluorescence angiography interpretation
Source: Surg Endosc. 2022 May 11;36(12):8764–73. doi: 10.1007/s00464-022-09299-3 (PMC9652172; doi:10.1007/s00464-022-09299-3)
Supplement: Supplementary file 1 — Supplementary file1 (DOCX 13 kb) [file 464_2022_9299_MOESM1_ESM.docx]

**Supplementary Information:** Questions asked during the interviews with the expert ICGFA users.

**Computer Assisted ICG Research Questionnaire**

Thank you for participating in our previous research on ICG. The paper has since been published.

We found some interesting differences in the ICG intensities and are hoping to follow up on how you made your choice of location for anastomosis in these specific videos. Please complete this quick survey and let us know your thoughts.

Please explain your philosophy or overall approach to how you select your transection point when using ICG in surgery?

Do you use ICG to guide your selection or confirm it?

What would you rank most (1) to least (6) important when using ICG around the time of transection point selection?

- The area is the site of greatest fluorescence on the screen
- The area is the point of distinction between fluorescence and non-fluorescence
- The area is the first place on the screen to light up
- The area of fluorescence corresponds to the site of mesocolic transection
- The area fluoresces homogenously with the rest of the perfused area
- The area gains fluorescence over a specific duration of time

Particularly when you started using ICG did you ever need to run the video a second time or give a second dose because you missed or were unhappy with the fluorescence?

Any additional feedback?

Thank you for your time and additional contribution towards this research, we value you the effort you put in to improve our understanding and optimisation of ICG in surgery.
